# Adherence to exercise and fitness following exercise-based outpatient cardiac rehabilitation: a cross-sectional survey for Germany

**DOI:** 10.1186/s13102-022-00585-0

**Published:** 2022-11-08

**Authors:** Nina Tilgner, Dominik Nehls, Celine Lichtmess, Alexandra Kober, Cinja Küsel, Lisa Radloff, Lars Gabrys

**Affiliations:** University of Applied Sciences for Sport and Management Potsdam, Potsdam, Germany

**Keywords:** Cardiac rehabilitation, Secondary prevention, Adherence, Compliance, Participation, Exercise, Physical activity

## Abstract

**Background:**

Exercise-based cardiac rehabilitation is safe and effective, evidence-based and implemented in national and international cardiac rehabilitation guidelines. Recent data show a decrease in cardiovascular mortality, reduced hospital admissions and an overall improvement in quality of life. To maintain positive effects and to prevent further cardiovascular events a major goal of cardiac rehabilitation is to induce a long-term health behaviour change and the integration of regular physical activity and exercise training in everyday life. The aim of this study is to evaluate the adherence of cardiac patients to exercise-based programs following rehabilitation phase III.

**Methods:**

A nationwide online cross-sectional survey was conducted. All outpatient aftercare providers who offer sports rehabilitation programs (heart groups) for cardiac patients in Germany were contacted. The questionnaire comprised 15 questions in five subcategories (general information regarding the outpatient aftercare provider, structure of rehabilitation sport programs, membership structure, content of heart groups, adherence to exercise-based programs).

**Results:**

560 of 2447 outpatient aftercare providers participated in the survey (response rate: 23%). On average, rehabilitation sport facilities hosted 2 (IQR 2) heart groups per week, and 23 patients (IQR 30) (61% males; 31% females) per facility completed rehabilitation sport prescription in 2018. Almost all providers offer follow-up programs on a self-payer basis after rehabilitation sport prescription ends. Adherence to follow-up programs was at 54% (IQR 65; 55% males and 50% females). With 60% (IQR 71), patients with a statutory health insurance (mainly pensioners) adhere slightly more often to a follow-up program compared to privately insured persons (mainly population with a high income or civil servants) with 50% and significantly more often compared to persons who were insured by the German pension fund (covering working population) with only 9% (IQR 89) adherence.

**Conclusion:**

Almost all outpatient aftercare providers offer follow-up programs for cardiac rehabilitation patients but only half of them actually participate. Younger people (working population) do not adhere sufficiently to sport and exercise programs following rehabilitation phase III. This seems critical to address in terms of achieving long-term rehabilitation goals.

## Introduction

Exercise-based cardiac rehabilitation is safe and effective, evidence based and implemented in national and international cardiac rehabilitation guidelines [1] and health care [2]. Recent data show a decrease in cardiovascular mortality, reduction in hospital admissions and an overall improvement of quality of life [3, 4]. To maintain these positive effects and to prevent further cardiovascular events a major goal of cardiac rehabilitation is to induce a long-term health behaviour change and an integration of regular physical activity and exercise training in everyday life. The European guidelines on cardiovascular disease prevention [5] highlight physical activity as a major goal for cardiovascular prevention. The guidelines recommend *“at least 150 min a week of moderate aerobic PA … or 75 min a week of vigorous aerobic PA. or a combination thereof.”* This is in line with the “Global recommendations on physical activity for health” from the World Health Organization (WHO) [6].

Coronary heart disease and myocardial infarction are major health problems in Germany with a lifetime prevalence of 5.7% in women and 10.4% in men [7]. According to WHO data, ischaemic heart disease is the number one cause of death worldwide [8].

After a cardiac event, the rehabilitation process within the German health care system comprises three different rehabilitation stages: first stage (phase I) focuses on intensive care and early mobilisation in the hospital. The second stage (phase II) includes a 3–4 week in- or outpatient cardiac rehabilitation program with a focus on improving physical capacity, disease management and improving quality of life [9]. The goal of rehabilitation phase II is to reintegrate the patient into the working process or daily life, respectively. To a large extent cardiac rehabilitation phase III consists of regular exercise-based outpatient aftercare rehabilitation programs. In these group-based exercise programs (heart groups) the patients exercise regularly to improve physical capacity and reduce cardiovascular risk. The described concept of rehabilitation sports usually comprises exercises to improve body conception, functional gymnastics, strength and endurance training, as well as educational measures to integrate physical activity into daily routine [10, 11].

In Germany, every patient has the right to participate in these structured exercise-based outpatient aftercare programs. Only skilled rehabilitation trainers who must be licensed and listed either by the German Society of Cardiovascular Prevention and Rehabilitation (DGPR) or the German Handicapped Sports Association (DBS) can offer these “heart groups”. Patients should exercise 1–2 times a week for at least 60 min. under surveillance of a physician. Heart group participation is usually prescribed by a physician and costs are covered by social insurance agencies. A prescription typically comprises 90 training sessions within 24 months.

Depending on age and working ability of the patient either (statutory) health insurances, the German pension fund or private health insurances (e.g. patients with a higher income, self-employed, civil servants) have to pay for this service. For the majority of the working population the German pension fund and for pensioners the (statutory) health insurances cover rehabilitation costs.

According to data of the DGPR some 9000 heart groups with a total of about 180,000 cardiac patients exist in the German outpatient aftercare setting [12].

What is so far unknown is how many patients continue with regular physical training following rehabilitation phase III, after prescription and cost coverage for rehabilitation sport ends.

Therefore, the aim of this study is to evaluate the long-term adherence of cardiac patients to exercise-based programs following rehabilitation phase III.

## Methods

### Participants

The study was conducted as a nationwide cross-sectional online survey among outpatient aftercare providers for rehabilitation sports. For this reason, all available e-Mail addresses of outpatient rehabilitation providers in Germany who are certified and listed at the DGPR or DBS websites were systematically collected. The analyses are based on aggregated data which were provided by the aftercare providers. No individual data was collected.

### Questionnaire

The questionnaire comprised 15 questions and was grouped in five subcategories. (1) General information about the outpatient aftercare provider (2) structure of rehabilitation sport programs (3) membership structure (4) content of heart groups and follow-up programs (5) adherence to follow-up programs. The main focus of the questionnaire was to analyse the adherence of cardiac patients to follow-up programs. For this reason, all providers were asked how many of their patients did participate in a sport or fitness program after prescription for rehabilitation sports (heart groups) ends in 2018.

The questionnaire was implemented in the online software SosciSurvey [13]. Prior to the main survey, a pre-test (2 weeks) was conducted to test comprehension, consistency, and filtering of the online questionnaire. Additionally, two independent experts in the field of cardiac rehabilitation reviewed the content and feasibility of the questionnaire.

The main survey was rolled out on January 7th, 2020. All outpatient aftercare providers who offer sports rehabilitation programs (heart groups) for cardiac patients in Germany were contacted via email and were invited to participate in the survey. The survey was online for 45 days. Three reminders were sent via E-Mail to increase participation rate.

### Statistical analysis

Data were collected electronically via SosciSurvey [13] and checked for plausibility. Due to the data not being normally distributed, non-parametric test procedures (Kruskal Wallis Test) were used for group comparisons. A multivariable logistic regression model was applied to test for a relationship between overall adherence to exercise programs (dependent variable) and the size of the outpatient-aftercare providers (number of members), diversity of follow-up (number of different follow-up programs) and costs of the follow-up programs as independent variables. All analyses were performed with the statistical software package SPSS (IBM SPSS Statistics 25). *p* < 0.05 was considered to indicate statistical significance.

## Results

Overall, 560 of 2447 outpatient aftercare providers participated in the survey (response rate: 23%). On average, rehabilitation sport facilities provided 2 (IQR 2) heart groups per week and most facilities (70%) offer rehabilitation sport programs for other diseases in addition to cardiovascular diseases. For descriptive analysis see Table 1.

**Table 1:** Characteristics of outpatient aftercare providers for exercise base cardiac rehabilitation who participated in the survey (N = 560)

Characteristic	Outpatient- aftercare providers
Contacted providers [N]	2447
Response rate [N, %]	560 (23)
Number of members per provider [median, IQR]	621 (1286)
Provider foundation [yrs. ± SD]	25 ± 12
Number of “heart groups” per week [median, IQR]	2 (2)
Other rehabilitation sports groups (e.g. orthopaedic or metabolic disease) [ %]	70

With 96%, most outpatient aftercare providers indicate gymnastics as a main training aspect of their heart groups, followed by relaxation/stretching (87%) and endurance training/games (86%).

### Structure of the follow-up programs

Almost all (98%) outpatient aftercare providers who participated in the survey offer follow-up programs after prescription for rehabilitation sport ends. These follow-up programs are offered on a self-payer basis and address mainly (85%) the same aspects as the heart groups (e.g. gymnastics, endurance training). On average, providers offer four different (IQR 3) exercise-based follow-up programs for heart patients. These include: gymnastics, endurance training, relaxing/stretching, strength training or bicycle ergometry. Costs for follow-up programs were 14 ± 13 € (mean ± SD) per month.

### Completion of prescription and adherence to exercise-based follow-up programs

On average 23 (IQR 30) cardiac patients (61% men) per outpatient aftercare provider completed rehabilitation sport prescription in 2018. The overall adherence to sport and exercise in terms of participation in a follow-up program was 54% (IQR 65) after prescription and cost coverage for rehabilitation sport ends with no sex specific difference. Whereas adherence to a follow-up program according to health insurance status of the patients show large differences between the insurance groups. Potentially younger, working patients whose costs for rehabilitation sport were covered by the German pension fund (GPF) show the lowest adherence to sport and exercise with an average participation rate of 9%. Rather older and non-working persons (mostly pensioners) whose costs were covered by statutory health insurances (SHI) or a private health insurance (PHI) show significantly higher participation rates of 60% and 50%, respectively compared to the GPF group. Detailed results are shown in Table 2.

**Table 2:** Adherence to sport and exercise according to health insurance status of cardiac patients who completed rehabilitation sports prescription in 2018 (N = 279)

insurance status [N = 99]	[%] median (IQR)	test statistic	Standard error	*p*-value**
statutory health insurance (SHI)	60 (71)			
private health insurance (PHI)	50 (100)			
German pension fund (GPF)	9 (89)			
*GPF –PHI		− 62.5	15.7	0.000
*GPF-SHI		− 87.2	14.5	0.000
*PHI-SHI		24.7	15.5	0.331

*Kruskal Wallis test for group comparison

**Significance level 0.05 with Bonferroni-correction

The multivariable logistic regression analysis to test for potentially predictive factors (outpatient aftercare provider size, number of follow-up programs, costs) affecting the overall adherence revealed no association between dependent and independent variables (R^2^ = 0,034, *p* = 0.537). Effect measures for outpatient aftercare size were OR 1.00 (95% CI 1.00–1.00) for number of follow-up programs OR 0.95 (95% CI 0.76–1.18) and costs OR 0.99 (95% CI 0.97–1.02).

## Discussion

The main finding of this study is, that even though almost all outpatient aftercare providers offer follow-up programs for cardiac rehabilitation patients, only half of the patients are successfully transferred to those programs after prescription and cost coverage ends. It also means that only half of the cardiac rehabilitation patients are transferred to long-term activation in terms of adherence to physical activity and exercise.

Next to an overall low adherence, our data show a huge difference in the participation in follow-up exercise-based programs according to the patients’ insurance status. The Germany pension fund covers the costs for most of the working population (usually ≤ 66 years) and the statutory health insurances cover the costs for most of the older non-working (retired) population. The huge difference in the adherence to sport and exercise programs between the insurance and age groups may be partly explained by a lack of time in the working population (pension fund adherence was at 9% vs. statutory health insurance adherence was 60%) and a probably not adequate program structure for the target group. So far, previous studies mostly investigated early drop out and non-attendance in phase II rehabilitation. Gaalema et al. [14] identified current smoking, lower socioeconomic status, younger age and non-surgical diagnosis as robust predictors for lower attendance rates whereas older age (65 years and older) was a strong predictor in higher attendance of cardiac rehabilitation sessions [14]. These results are in line with our results of low attendance rates in younger age groups. Another study of Mikkelsen et al. [15] came to a similar conclusion that (younger) age, family status and employment play a major role in non-attendance in cardiac rehabilitation due to lack of time [15]. As described before, there are only a few studies available which focus on predictors for successful adherence to cardiac rehabilitation in phase III and many of those studies are not directly comparable because of differences in program content and design [16].

Wieczorrek et al. found similar results according to the overall adherence of cardiac rehabilitation phase III as we did. They compared a standard heart group to a Tai-Chi based program. The adherence in both groups was similar, with 50% for the Tai-Chi based program and 48% for the standard heart group [17]. In the CARO II study (Cardiac Rehabilitation Outcome) Dohnke et al. analysed the correlation of (low) participation and motivation in rehabilitation phase III by the HAPA model (health action process approach) [18]. The HAPA model ranks patients as non-intenders, intenders or actors by differences in social-cognitive factors via questionnaires. In the CARO II study results, 6 and 12 months after rehabilitation phase II revealed 56% non-intenders, 13% intenders and 31% actors. The authors concluded that the psychological state of the patients seems to play a major role in their participation and their motivation for long-term program adherence and therefore should be considered in secondary prevention.

To achieve long-term change of health behaviour in cardiac patients and maintain and improve quality of live adherence to regular physical activity is necessary [3, 4]. This is well described in national S3 guidelines for cardiac rehabilitation [1] as well as in the 2020 ESC European guidelines on sports cardiology and exercise [19]. The German concept of exercise-based heart groups in the rehabilitation phase III is a cornerstone of the effort to achieve this goal. Based on the guidelines exercise-based cardiac rehabilitation (phase III) should address different types of activities e.g. exercises improving body conception, functional gymnastics, strength and endurance training, coordination and the recommendation to be physically active doing self-organized exercise [20]. These contents match with the results of our study. Almost all heart group providers offer activities according to the guidelines like gymnastics and/or endurance training. Price et al. reviewed different international guidelines for cardiac rehabilitation exercise programs and concluded that aerobic endurance training is the basis of exercise therapy in cardiac rehabilitation, but the intensity varies between national recommendations, and resistance training should be implemented more often, as well [21]. A study of Gabrys et al. recently analysed the adherence to phase III cardiac rehabilitation programs in association to mortality and working capacity. Patients who participated in exercise-based phase III rehabilitation programs showed reduced mortality rates and a reduced loss in working capacity [22].

One advantage for future exercise-based cardiac rehabilitation programs could be a more personalized approach [23, 24] e.g. a gender-specific program structure to achieve better acceptance and higher adherence rates in the target group [25, 26]. Results indicate a better acceptance in women who participate in special women programs compared to mixed gender programs [27, 28]. Maybe this could also improve adherence to exercise-based secondary heart prevention in women.

According to our analysis we could not find any predictive factors of outpatient aftercare provider structure influencing the adherence to exercise and fitness in the target group.

A loss of almost 50% of cardiac patients after cost coverage for rehabilitation sport ends seems critical in terms of achieving long-term rehabilitation goals. Especially when we know that only a minority of 9.7% to 22.5% of cardiac patients are successfully referred to rehabilitation sport programs (heart groups) following rehabilitation phase II [29].

## Strengths and limitations

A strength of the study is that all outpatient aftercare providers who offer heart groups for cardiac patients in Germany were identified and invited to participate in the survey. This is as far as we know the largest survey among outpatient aftercare providers in Germany.

Unfortunately, our data give no information regarding whether patients exercise in self-organized or home-based programs. Data was collected on an aggregated level from outpatient aftercare providers, and we have no information about participants on an individual basis. The survey was filled out by employees of the outpatient aftercare providers. Therefore, information regarding predictive factors of (low) participation rate is limited to external factors of the outpatient aftercare providers and not available for each individual separately.

Further research using individual data is needed to investigate predictive factors and to adapt existing programs in order to improve adherence to exercise-based cardiac rehabilitation programs. To address these limitations a nationwide patient-based survey would be the method of choice. Health insurance companies usually ask their patients about their satisfaction with the rehabilitation process from time to time. This could be a good starting point for future collaborative health service research.

## Conclusion

Although almost all outpatient-aftercare providers who completed the survey (response rate: 23%) offer follow-up programs for cardiac rehabilitation patients, only half of them actually participate. In particular, younger patients do not adhere sufficiently to sport and exercise following rehabilitation phase III. This seems critical in terms of achieving long-term rehabilitation goals in this population.

## Data Availability

The dataset used and analysed during the current study is available from the corresponding author on reasonable request.

## Abbreviations

DBS: German Handicapped Sports Association
DGPR: German Society of Cardiovascular Preventions and Rehabilitation
GPF: German pension fund
PHI: Private health insurance
SHI: Statutory health insurance
